# Spatiotemporal Differentiation and Driving Force Analysis of the High-Quality Development of Urban Agglomerations along the Yellow River Basin

**DOI:** 10.3390/ijerph19042484

**Published:** 2022-02-21

**Authors:** Yu Chen, Qianqian Miao, Qian Zhou

**Affiliations:** 1 School of Economics and Management, Zhengzhou University of Light Industry, Science Avenue 136, Zhengzhou 450000, China; 2012030@zzuli.edu.cn (Y.C.); miaoqianqian0301@163.com (Q.M.); 2 Economics School, Zhongnan University of Economics and Law, Nanhu Avenue 182, Wuhan 430073, China

**Keywords:** high-quality development (HQD), spatiotemporal differentiation, geographically-weighted regression (GWR), urban agglomerations, Yellow River Basin (YRB)

## Abstract

The ecological protection and high-quality development (HQD) of the Yellow River Basin (YRB) have been promoted as national strategies. An urban agglomeration is the basic unit of the YRB used to participate in international competitions. Taking seven urban agglomerations covering 70 cities along the YRB as the sample, this paper establishes a high-quality evaluation system and uses the entropy method and exploratory spatial data analysis (ESDA) to analyze the HQD levels of the seven urban agglomerations along the YRB from 2009 to 2018. In addition, geographically-weighted regression (GWR) is adopted to analyze the influencing factors. The results show that: (1) the gap in the HQD of the seven urban agglomerations gradually narrows, showing a spatial pattern of “high in the east, low in the west, and depression in the middle”; (2) the HQD levels of the seven urban agglomerations have a strong spatial correlation, and the patterns of cold and hot spots have not changed substantially, showing the spatial distribution of “hot in the east, cold in the west”; (3) the degree of influence of each driving factor on the HQD differs among the seven urban agglomerations. The order is as follows: industrial structure upgrading index > proportion of R&D expenditure > urbanization rate > internet penetration rate > proportion of urban construction area > proportion of days reaching the air standard. These findings show that advanced industrial structure and technology are the two core driving forces for the HQD of the urban agglomerations along the YRB.

## 1. Introduction

The YRB is an important ecological barrier and economic zone in China, and it is also a key area used to promote regional coordinated development [1]. The urban agglomerations along the YRB comprise three regional urban agglomerations (Shandong Peninsula Urban Agglomeration, Central Plains Urban Agglomeration, and Guanzhong Plain Urban Agglomeration) and four local urban agglomerations (Lanxi Urban Agglomeration, Jinzhong Urban Agglomeration, Hohhot-Baotou-Ordos-Yulin Urban Agglomeration, and Ningxia Urban Agglomeration along the Yellow River), which together form a “3 + 4” spatial organization pattern [2]. Over 70% of the population and 80% of the total economy of the YRB are concentrated in 33% of the land area of the urban agglomerations [3]. However, urban agglomerations also emit more than 70% of pollution, thus representing a serious disaster area regarding environmental pollution. Moreover, the proportion of GDP in urban agglomerations decreased significantly after 2012. Therefore, the lagging economic development, local environmental pollution and high ecological potential risk are the three major problems faced in the HQD of the YRB [4]. In this regard, ecological protection and HQD in the YRB were proposed as major national strategies in September 2019, and the subsequent meetings further emphasized the promotion of HQD in central cities and urban agglomerations along the YRB. Based on this, this paper attempts to explore the specific driving factors for the improvement of the HQD of urban agglomerations in the YRB from the spatial evolution of the HQD level in the past decade to the extent that these driving factors affect the HQD level of urban agglomerations. Moreover, is the effect the same in different areas of the YRB? In order to answer the above issues, this paper takes seven urban agglomerations along the YRB covering 70 major cities from 2009 to 2018 as the research sample. We start from the new development concepts of “innovation, coordination, greening, opening and sharing” to investigate the spatiotemporal differentiation pattern of the HQD of seven urban agglomerations along the YRB. In addition, we select six driving factor indicators, namely, population, industry, innovation, internet, urban construction and ecological environment, and use GWR to deeply analyze the factors affecting HQD. This study is expected to provide a targeted reference for the accurate, coordinated and efficient development of seven urban agglomerations along the YRB.

Internationally, research on “urban agglomerations” began at the end of the 19th century and considered “single city” to “urban agglomerations”. In the late 19th century, Ebenezer Howard, a British urbanist, proposed the concept of an “urban cluster” in his book, *Garden Cities of Tomorrow*; this concept was regarded as the germ of the idea of urban agglomeration [5]. The “urban cluster” concept changed the original spatial focus on a single city to the study of the spatial area comprising multiple surrounding garden cities. The relationship between edge cities and neighboring cities is more independent, and it is a concentrated area dominated by employment. Suburbanization is a process in which population, industry and commerce, and service industry are gradually transferred from cities to suburbs, and an agglomeration economy is generated. In 1915, P. Geddes, a British sociologist, suggested that based on evolutionary research of urban elements and their spatial and industrial layout that the existing urban expansion was the result of the excessive separation between urban and suburban areas and that urban concentration would be the trend of urbanization in the future. Therefore, he proposed the concept of conurbations or urban agglomerations [6]. In 1957, geographer Jean Gottman coined the name megalopolis for an urban agglomeration for the first time and clearly pointed out that the economic form of dominating space in the future is no longer a single city but a megalopolis area formed by regional integration, namely, an urban agglomeration [7]. Therefore, Jean Gottman is considered the main contributor to the study of urban agglomerations, and an increasing number of scholars have paid attention to this topic. In the 1980s, Chinese urban geographers became the first research group to pay attention to the problem of “urban agglomerations” in China. Based on Jean Gottman’s research idea, they pointed out that urban agglomerations are the aggregation of multiple cities with the core of the central city radiating to the surroundings [8].

The term “HQD” was first proposed at the 19th National Congress of CPC in 2017 [9], indicating that China’s economy has shifted from “high-speed” to “high-quality” development. To this end, relevant scholars have closely followed the situation of Chinese economic development and interpreted its connotation of HQD from different perspectives. From the perspectives of “quantity” and “quality”, some scholars believe that HQD means that economic development no longer simply pursues the expansion of quantity but that quantity and quality are increased simultaneously and succeeds by increasing the quality; this concept is considered the coordinated development of quantity and quality [10]. From the perspective of macroeconomics, some scholars have pointed out that HQD is a sustainable development strategy with fewer production factor inputs, high-efficiency resource allocation, low resources and environmental costs, and good economic and social benefits [11]. In addition, some scholars have combined macroeconomics, industrial development and enterprise management and proposed a fully balanced concept of development that can provide comprehensive and systematic strategic guidance for Chinese economic development [12].

Evaluating the HQD of urban agglomerations is the basis for exploring the development differences and achieving coordinated development. In the evaluation of HQD research, the composite index is more convincing than the single index; therefore, the current research aims to build a composite index evaluation system [13]. According to different research areas and perspectives, composite indicators are mainly established from the following two aspects. Early research focuses on the economic benefits of urban development and constructs an evaluation system from the four dimensions of population, economy, society and ecology [14,15]. The current research is mostly guided by the new development concept and constructs the HQD evaluation system from the five dimensions of innovation, coordination, green, opening and sharing [16,17]. Since the quality of urban agglomeration development is directly linked with the development concept, the evaluation system has been widely recognized by the academic community.

Clarifying the core driving factors of the HQD of urban agglomerations is the key to the accurate positioning and scientific development of urban agglomerations [18]. To date, there have been few studies on the driving factors of HQD in urban agglomerations, and these studies have mainly focused on the two aspects of development elements and the development environment. Specifically, development elements, such as human capital [19], R&D investment [20], industrial development, informatization level [21], transportation infrastructure, and foreign investment level [22], have significantly promoted urban agglomeration development. A higher development environment level, such as that characterized by the per-capita GDP, opening up [23], scientific and technological services, advanced industrial structure [24], urbanization, and consumption capacity [25], is more conducive to the realization of a higher urban agglomeration development level.

In summary, the interpretation of the connotation of urban was relatively mature and has laid solid theoretical foundations for this research. Regarding the evaluation systems of HQD, some experts and scholars have carried out diversified demonstrations based on different perspectives. Unfortunately, a unified research system has not yet been formed. Few studies have focused on the driving factors of HQD, and there is a lack of research on regional differences. Compared with the existing literature, the innovations and contributions of this paper are as follows: (1) In terms of scale, breaking the limitations of previous independent urban agglomeration and provinces as urban clusters, this paper studies multiple urban agglomerations from the level of prefecture-level cities. (2) In terms of research methods, ESDA analysis is used to quantitatively study the evolution of the spatial pattern of HQD in urban agglomerations. The GWR model is used to seek the path of HQD, which makes up for the shortcomings of the lack of discussion on the internal differences of urban agglomerations in previous studies. (3) In terms of influencing factors, in addition to economic, social and social drivers, this paper also adds ecological environment drivers, comprehensively considering the influencing factors of HQD of urban agglomeration along the YRB.

The rest of the research is as follows: the second section introduces the research area, research methods and the evaluation index system; the third section analyses the overall and local characteristics of the HQD of urban agglomerations in the YRB from the perspective of time and space, focusing on the reasons for the spatial heterogeneity affecting the HQD of urban agglomerations. Finally, the conclusions are drawn, and the recommendations to improve the HQD of urban agglomeration in the YRB are given.

## 2. Research Areas and Methods

### 2.1. Research Areas

According to the national 13th Five-Year Plan, there are 7 urban agglomerations along the YRB. The upper, middle and lower reaches of the Yellow River are defined according to the division scheme of the Yellow River Water Conservancy Commission. The two dividing points are Hekou town in Tuoketuo County and Taohuayu in Zhengzhou. Considering the confluence area, the Hohhot-Baotou-Ordos-Yulin Urban Agglomeration is categorised into the upstream region, while the Central Plains Urban Agglomeration is located downstream. From upstream to downstream, the river flows through the central cities of Lanzhou, Yinchuan, Hohhot, Taiyuan, Xi’an, Zhengzhou and Jinan. The upper reaches include the Lanxi Urban Agglomeration, Ningxia Urban Agglomeration, and Hohhot-Baotou-Ordos-Yulin Urban Agglomeration; the middle reaches comprise the Jinzhong Urban Agglomeration and Guanzhong Urban Agglomeration; and the lower reaches contain the Central Plains Urban Agglomeration and Shandong Peninsula Urban Agglomeration. After careful consideration, we selected 70 cities in the 7 urban agglomerations as the research sample.

### 2.2. Research Methods

#### 2.2.1. Entropy Method

In the comprehensive evaluation index system, due to the different functions and influence degrees of each index, the weight of each index should be assigned according to its importance. Therefore, we first use the “Mini-max standard method” to process the data and eliminate the dimensionality effect among indexes. Then, we use the entropy method to objectively determine the weight of each index. The formula is as follows:

(1) $$P_{\mathrm{ij}}=\frac{X^{\prime }{}_{\mathrm{ij}}}{\sum_{i=1}^{n}X_{\mathrm{ij}}}\;e_{j}=-\frac{1}{\mathrm{lnn}}\sum_{i=1}^{n}p_{\mathrm{ij}}\ln\left(p_{\mathrm{ij}}\right)$$

where $P_{\mathrm{ij}}$ is the proportion of index J in region I (reflected by the average value of cities in the region); $X^{\prime }{}_{\mathrm{ij}}$ is the standardised matrix; $X_{\mathrm{ij}}$ is the original matrix; $e_{j}$ is the entropy value, with 0 ≤ ${\;e}_{j}\;$ ≤ 1; and n is the number of urban agglomerations, n = 7.

(2) $$g_{j}=1-e_{j}\;w_{j}=\frac{g_{j}}{\sum_{i=1}^{m}g_{j}}$$

where $g_{j}$ is the standard coefficient; $w_{j}$ is the weight of each indicator; and m = 23 is the number of evaluation indicators.

(3) $$s_{i}=\overset{m}{\underset{j=1}{\sum }}w_{j}\times p_{\mathrm{ij}}$$

where $s_{i}$ is the comprehensive score of the HQD level in region I.

#### 2.2.2. Exploratory Spatial Data Analysis

In this paper, we perform exploratory spatial data analysis (ESDA) to reveal the spatial association characteristics between geographical units and explore the spatial heterogeneity of research objects. Then, we use the global ${\mathrm{Moran}}^{’}s\;I$ index and $\mathrm{Getis}-{\mathrm{Ord}\;G}_{i}^{*}$ index to analyse the global and local spatial autocorrelation of the HQD level of urban agglomerations.

The global ${\mathrm{Moran}}^{’}s\;I$ index can reflect the similarity of the attributes of adjacent areas, which reveals the spatial correlation and spatial dependence of the whole research area. The mathematical expression is as follows:

(4) $${\mathrm{Moran}}^{’}s\;I=\frac{\sum_{i=1}^{n}\sum_{j=1}^{n}W_{\mathrm{ij}}\left(Z_{i}-\;\bar{Z}\right)\left(Z_{j}-\;\bar{Z}\right)}{S^{2}\sum_{i=1}^{n}\sum_{j=1}^{n}W_{\mathrm{ij}}}$$

where n is the sample size and $Z_{i}$ and $Z_{j}$ represent the observed values of the HQD index in region i and region j, respectively; $\bar{Z}$ is the mean value; $S_{2}\;$is the variance of the observed values; and $W_{\mathrm{ij}}$ is the spatial weight matrix. The value range of global ${\mathrm{Moran}}^{’}s\;I$ index is [−1, 1]. I < 0 indicates the spatial negative correlation, and there is a difference between adjacent regions; I > 0 indicates the spatial positive correlation, which shows that research units with high (low) attribute values show agglomeration in space; I = 0 indicates the lack of spatial correlation, which shows that units are randomly distributed in space.

Global spatial autocorrelation describes the average concentration and dispersion degrees of the whole study space, but it cannot describe the spatial location of the cluster in detail. In this paper, the $\mathrm{Getis}-{\mathrm{Ord}\;G}_{i}^{*}$ index is used to detect whether high-value spatial clustering (hot spot) or low-value spatial clustering (cold spot) occurs in local space. The formula is as follows:

(5) $$G_{i}^{*}=\overset{n}{\underset{j=1}{\sum }}W_{\mathrm{ij}}\frac{Z_{i}}{\sum_{j=1}^{n}Z_{j}}$$

where the meanings of relevant variables are consistent with Formula (4). $W_{\mathrm{ij}}$ is the spatial weight matrix, with 1 for spatial adjacency and 0 for non-adjacency. If $G_{i}^{*}$ is greater than 0, the spatial units around city i and the adjacent areas belong to the high-value agglomeration areas, namely, the hot spot areas. In contrast, it indicates that the spatial units around city i and the adjacent areas belong to the low-value areas, namely, the cold point areas.

#### 2.2.3. Geographically-Weighted Regression Model

The traditional linear regression model estimates all the samples and parameters globally without considering the spatial attributes of the data. The GWR is solved by the local weighted least square method, which is essentially local regression and can explore the spatial nonstationarity of parameters [26]. We use the latter to measure differences in influencing factors of HQD of 7 urban agglomerations along the YRB with the change in spatial location. The formula is as follows:

(6) $$y_{i}=\beta_{0}\left(u_{i},v_{i}\right)+\underset{j}{\sum }\beta_{j}\left(u_{i},v_{i}\right)x_{\mathrm{ij}}+\varepsilon_{i}$$

where $y_{i}$ is the dependent variable, $x_{\mathrm{ij}}$ is an independent variable (influencing factor), $\beta_{0}$($u_{i}$,$v_{i}$) is a constant term, ($u_{i}$,$v_{i}$) is the spatial coordinates of area i, $\beta_{j}\left(u_{i},v_{i}\right)$ is the variable parameter of the j-th explanatory variable $x_{\mathrm{ij}}$. in area i, and $\varepsilon_{i}$ is the random error term.

### 2.3. Index System Construction and Data Source

China’s economy has entered a critical period of rapid shift, structural adjustment and kinetic energy conversion [27]. The evaluation index system of the HQD of urban agglomerations should be based on the new development concept of “innovation, coordination, greening, opening and sharing”. Innovation is the core of HQD [28]. Based on the consideration of innovation inputs and outputs, we select three indicators to measure: the proportion of science in the general public budget expenditure, the proportion of education in the general public budget expenditure, and the number of patents granted per 10,000 people. Coordination is the guarantee for HQD [29]. Starting from the perspectives of urban and rural areas and regions, this paper takes the coordination of income and consumption as the measurement standard to comprehensively evaluate the coordinated development among cities and urban agglomerations. Greening is an inevitable requirement for HQD [30], especially in the YRB, which is rich in resources but has a fragile ecological environment. This paper establishes a green development index system from the three aspects of urban greening, pollution emissions and green governance. Opening development is a basic national policy of China [31], and has become the external window of HQD. This paper constructs an opening development system from three perspectives: trade opening, investment opening and tourism opening. Sharing is the fundamental principle of high-quality social development [32], urban roads and public transportation, which are the cornerstones of people’s sharing, medical security is the guarantee of sharing, and cultural sharing is the sublimation of daily life, which together reflect a more comprehensive HQD.

This paper takes 70 cities as the basic research unit of 7 urban agglomerations along the YRB during 2009–2018. The data come from the China Statistical Yearbook, China City Statistical Yearbook, China Statistical Yearbook for Regional Economy, EPS database, statistical yearbooks of relevant provinces and cities, statistical bulletins of national economic and social development, etc. All the data are filtered one by one to remove outliers. For some missing data, we use the interpolation method to make up and ensure the continuity of the data. According to the connotation and evaluation system of HQD, referring to the results of previous studies, following the principles of scientific, comprehensive, representative and operable index system construction, we established the HQD evaluation index system composed of 23 specific indicators in 5 dimensions of innovative development, coordinated development, green development, opening development and sharing development, as shown in Table 1.

## 3. Results

### 3.1. Analysis of the Spatiotemporal Pattern of HQD in Urban Agglomerations

#### 3.1.1. Characteristics of Spatiotemporal Variations

**Figure 1:**
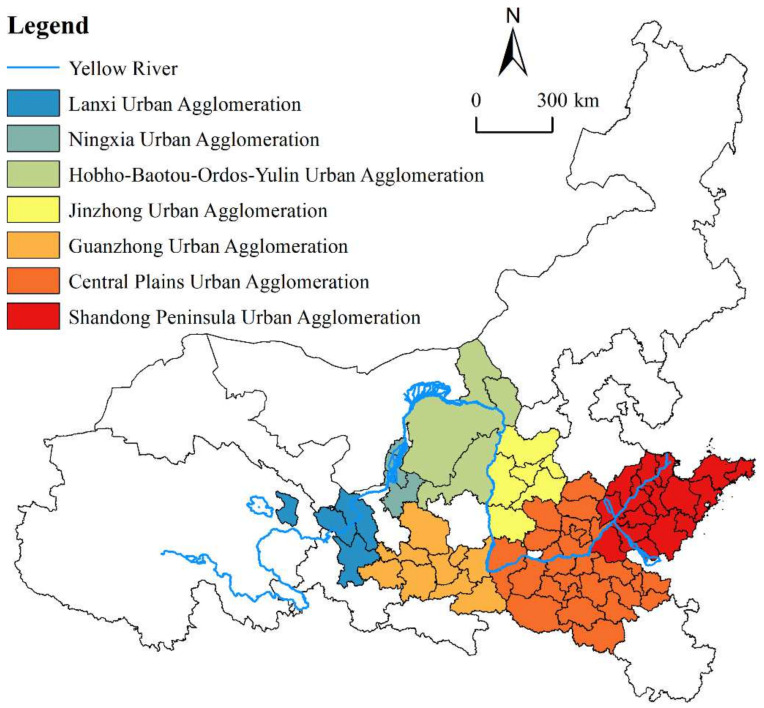
Research areas.

**Figure 2:**
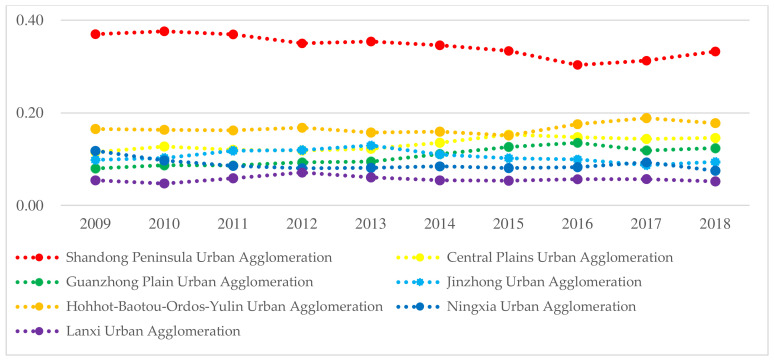
Temporal change in the HQD level of seven urban agglomerations.

According to Formulas (1)–(3), we calculate the HQD index of seven urban agglomerations along the YRB from 2009 to 2018, analyze the temporal trend (Figure 1), and generate the spatial distribution pattern in 2009 and 2018 with the help of ArcGIS 10.2 (Figure 2).

(1) Temporal variation characteristics

In the past 10 years, the HQD level of the Shandong Peninsula Urban Agglomeration has been far ahead. The gap among the seven urban agglomerations decreased from 0.32 in 2009 to 0.28 in 2018. With this gap gradually narrowing, the coordinated development of the YRB was significantly enhanced. From the changing trend, the seven urban agglomerations present three change patterns. First, the HQD levels of the Central Plains Urban Agglomeration, Guanzhong Plain Urban Agglomeration and Hohhot-Baotou-Ordos-Yulin Urban Agglomeration show a fluctuating upward trend; second, the HQD levels of the Shandong Peninsula Urban Agglomeration, Jinzhong Urban Agglomeration and Ningxia Urban Agglomeration show a fluctuating downward trend; and third, the HQD level of the Lanxi Urban Agglomeration has shown almost no change. From the change range, the HQD index of the Guanzhong Plain Urban Agglomeration increases the most, followed by the Central Plains Urban Agglomeration. The HQD index of the Shandong Peninsula Urban Agglomeration and Ningxia Urban Agglomeration decrease the most. The change range of the high-quality development index of the other urban agglomerations is not obvious. Therefore, we decompose the five fractal dimension development indexes of each urban agglomeration and find that the Guanzhong Plain Urban Agglomeration and Central Plains Urban Agglomeration have benefited from the improvement of openness and sharing level. With the accelerated construction of the “Belt and Road” and “meter-shaped” high-speed rail networks and airports, the traffic conditions of the two urban agglomerations were significantly improved. Xi’an has become the forefront of the westward opening, and Zhengzhou Airport has become the gateway of opening to the outside world. The two central cities have actively played crucial roles in radiating and driving the development of the urban agglomerations to a higher level. However, the limitation of the innovation ability and the damage of green development have led to a significant decline in the Shandong Peninsula Urban Agglomeration and Ningxia Urban Agglomeration along the YRB.

(2) Spatial variation characteristics

**Figure 3:**
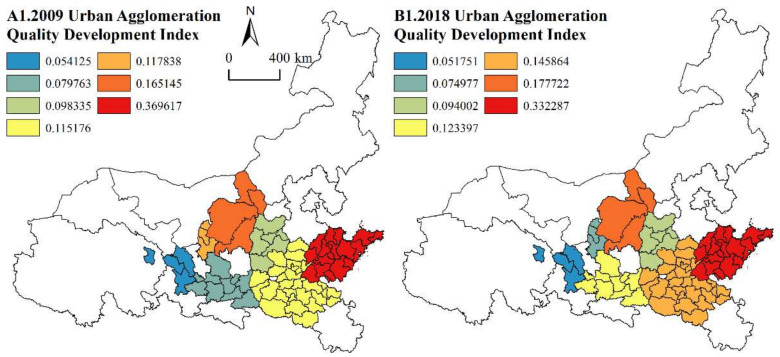
Spatial change in the HQD level of urban agglomerations in 2009 and 2018.

From Figure 3, we can see that the HQD levels of the seven urban agglomerations along the YRB present a spatial pattern of “high in the east, low in the west, and depression in the middle”. The eastern Shandong Peninsula Urban Agglomeration is the highest, the western Lanxi Urban Agglomeration is the lowest, and the central Guanzhong Plain Urban Agglomeration and Jinzhong Urban Agglomeration are low. According to the analysis of the five dimensions, the Shandong Peninsula Urban Agglomeration is high in all dimensions, while that of the Lanxi Urban Agglomeration is low in all dimensions. The HQD levels of the Guanzhong Plain Urban Agglomeration and Jinzhong Urban Agglomeration are limited by coordinated development and green development. Although the Guanzhong Plain Urban Agglomeration has Xi’an as the national central city, it lacks secondary central cities, and the development of small and medium-sized cities is relatively weak, which hinders the overall development of the urban agglomeration. The Jinzhong Urban Agglomeration vigorously develops the coal industry, which consumes many resources and causes serious environmental pollution. In the future, it should be guided by green development and rely on technological progress to promote green industrial development.

**Figure 4:**
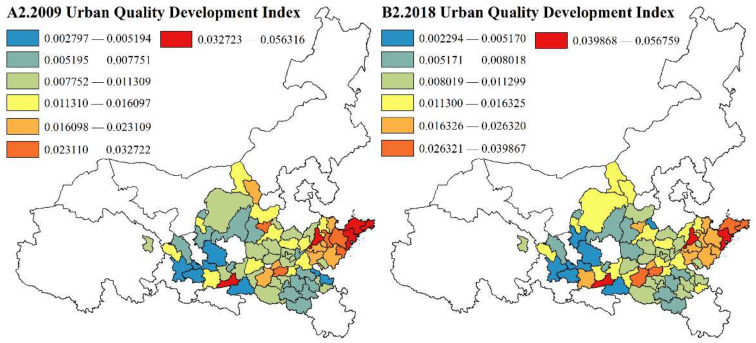
Spatial change in the HQD level of cities in 2009 and 2018.

From Figure 4, we can see that the HQD level of cities within each urban agglomeration along the YRB varies greatly, presenting an obvious “center-periphery” spatial pattern. The Central Plains Urban Agglomeration, Guanzhong Plain Urban Agglomeration, Jinzhong Urban Agglomeration, Ningxia Urban Agglomeration and Hohhot-Baotou-Ordos-Yulin Urban Agglomeration decrease from the single-core cities Zhengzhou, Xi’an, Taiyuan, Yinchuan and Hohhot to the besieged cities, the Shandong Peninsula Urban Agglomeration decreases from Jinan and Qingdao to the besieged cities, and the Lanxi Urban Agglomeration decreases from Lanzhou and Xining to the besieged cities. On the one hand, the core cities often have relatively developed transportation conditions and become the external connection hubs, and by creating good living and production environments, they have attracted high-end talent and strong enterprises to settle in, thereby driving the overall development of the corresponding region. On the other hand, the phenomenon also shows that core cities have a strong radiation driving effect on the development of surrounding cities. Developing core cities, cultivating secondary central cities and improving the development level of small- and medium-sized cities are important ways to improve the overall development of urban agglomerations along the YRB.

#### 3.1.2. Spatial Autocorrelation Analysis

(1) Global spatial autocorrelation analysis

**Figure 5:**
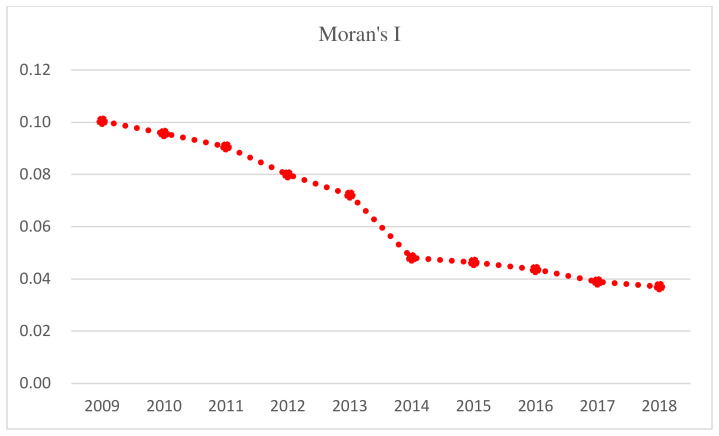
Moran’s I of HQD of seven urban agglomerations.

To quantitatively study the evolution of the HQD spatial patterns among the seven urban agglomerations, this paper establishes the Queen’s adjacency matrix and uses the data from 2009 to 2018 to calculate the global Moran’s I of the study area over the years (Table 2). The results show that the global Moran’s I index is greater than 0, the Z value is greater than 2.58, the *p*-value is less than 0.01, and the global Moran’s I index is significant at a 99% confidence level. These findings indicate that the HQD level of urban agglomerations along the YRB has a strong spatial correlation and an obvious agglomeration effect. That is, cities with HQD are concentrated together, and cities with low-quality development are concentrated and distributed. However, Moran’s I shows a downward trend (Figure 5), indicating that although the high-value (low-value) regions are still concentrated, the agglomeration is weakening, which is because, with the support of national policies, the central cities in the western region have developed rapidly. However, limited by the weak economic development foundation and fragile ecological environment, it is difficult for the central cities to produce diffusion effects and drive the surrounding cities to achieve HQD.

(2) Local spatial autocorrelation analysis

**Figure 6:**
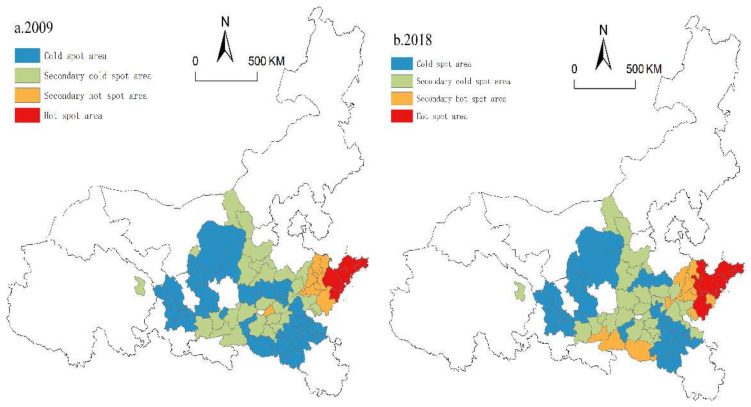
Spatial pattern of urban HQD in 2009 and 2018.

To eliminate the defect of global autocorrelation in covering up local instability and to detect the evolution characteristics of local spatial agglomeration patterns, this paper uses ArcGIS 10.2 to calculate the local $G_{i}^{*}$ index of each city in 2009 and 2018. We use the natural breakpoint method to divide the values into hot spot areas, secondary hot spot areas, secondary cold spot areas and cold spot areas, and the agglomeration evolution map of the urban HQD pattern along the YRB from 2009 to 2018 is drawn (Figure 6).

In general, the pattern of cold and hot spots in the HQD of urban agglomerations has not changed substantially, thus showing the characteristics of less hot areas and more cold areas and roughly showing the spatial distribution of “hot in the east and cold in the west”. The primary and secondary hot areas are mainly distributed in the Shandong Peninsula Urban Agglomeration. The remaining areas are mostly cold spots and secondary cold spots. This is because the Shandong Peninsula Urban Agglomeration has relatively high population quality and a clear innovation environment, which enables it to prioritize the development of high-end and green industries. Under the radiation of dual-core cities (Jinan and Qingdao), the overall development of urban agglomeration towards high quality was realized. In other areas, the development level of the central cities is mostly lower than that of Jinan and Qingdao; therefore, the radiation driving effect is limited, and these areas become low-value agglomeration areas.

In terms of the number of cold/hot spots, both cold spots and hot spots are shrinking; cold spots decreased from three cities in 2009 to 25 cities in 2018, and hot spots decreased from eight cities in 2009 to seven cities in 2018. The ranges of secondary cold spots and secondary hot spots are expanding: the secondary cold spot area increased from 23 cities in 2009 to 27 cities in 2018, and the secondary hot spot area increased from six cities in 2009 to 11 cities in 2018. From the spatial variation pattern of cold/hot spots, the cold spots in the Central Plains and Guanzhong Plain Urban Agglomerations have decreased, gradually transitioning to secondary cold spots and secondary hot spots because the rapid development of Zhengzhou and Xi’an has driven the development of Xuchang, Anyang, Jiaozuo, Xianyang, and Shangluo in the cluster while the upstream urban agglomerations lack hot cities to drive the HQD of the region. However, with the contraction of cold and hot spots and the expansion of secondary cold and hot spots, the spatial gap of HQD in seven urban agglomerations gradually narrows.

### 3.2. Research on the Spatial Differentiation of Driving Factors

#### 3.2.1. Driving Factor Analysis Framework

Realizing the HQD of urban agglomerations is complex, and current academia has not yet formed a unified view. Combining the existing literature, population size [33,34], industrial structure [35,36,37], science and technology [38,39], information level [40], urban construction [41,42], and environmental quality [43,44,45,46] have significant impacts on the HQD of urban agglomerations. Therefore, we select these six aspects as the driving factors that affect the HQD of seven urban agglomerations along the YRB (Table 3). Population urbanization is the starting point and foothold of the realization of “people-oriented” new urbanization. The upgrading of industrial structure is conducive to exerting industrial efficiency, which is an inevitable requirement to help the development of seven urban agglomerations. Innovation and technology can provide more innovative elements, which generate endless impetus for the development of the region, expressed by the proportion of R&D funds. The level of informatization is the basis of realizing smart cities, which can improve the overall efficiency of urban resource utilization expressed by the internet penetration rate. Urban construction is a barometer used to measure whether a region’s urbanization process is healthy and whether the quality of urbanization development is efficient, and it is expressed by the proportion of urban construction area. Environmental quality can directly reflect the level of regional ecological civilization construction and become the key to regional sustainable development, expressed by the proportion of days reaching the air standard. To analyze whether there was multicollinearity among the independent variables, we tested the six indicators (Table 4). The results show that the variance inflation factor (VIF) is less than 10, the tolerance (T) is greater than 0.1, and the conditional index (CI) is less than 30, indicating that there is no multicollinearity among the independent variables.

#### 3.2.2. Spatial Heterogeneity of Driving Factors

Previous papers have shown that there is a spatial correlation in the HQD of the seven urban agglomerations. Therefore, the GWR model can be further used to analyze the spatial differences of the influencing factors on the HQD of urban agglomerations. We set the core type to ADAPTIVE and choose the AICC as the bandwidth. The $R^{2}$ and adjusted $R^{2}$ of the OLS are 0.392 and 0.334, respectively, and the $R^{2}$ and adjusted $R^{2}$. of GWR and adjusted $R^{2}$ are 0.6363 and 0.5598, respectively. It shows that the GWR model has a better fitting effect and can better simulate the influence of various variables on the HQD of seven urban agglomerations.

The results show that the estimation results of each variable are different for the seven urban agglomerations, which indicates that there are spatial differences in promoting the HQD level of the seven urban agglomerations along the YRB. In terms of the sign of the regression coefficient, only the proportion of days with air reaching the target showed positive and negative effects, while all the other variables showed significant positive effects. From the mean value of the regression coefficient, the degree of influence of the six variables was ranked as follows: industrial structure upgrading index > proportion of R&D expenditure > urbanization rate > internet penetration rate > proportion of urban construction area > proportion of days reaching the air standard. This finding shows that advanced industrial structure and technology are the two core driving forces promoting the development of seven urban agglomerations along the YRB.

(1) Urbanization Rate

**Figure 7:**
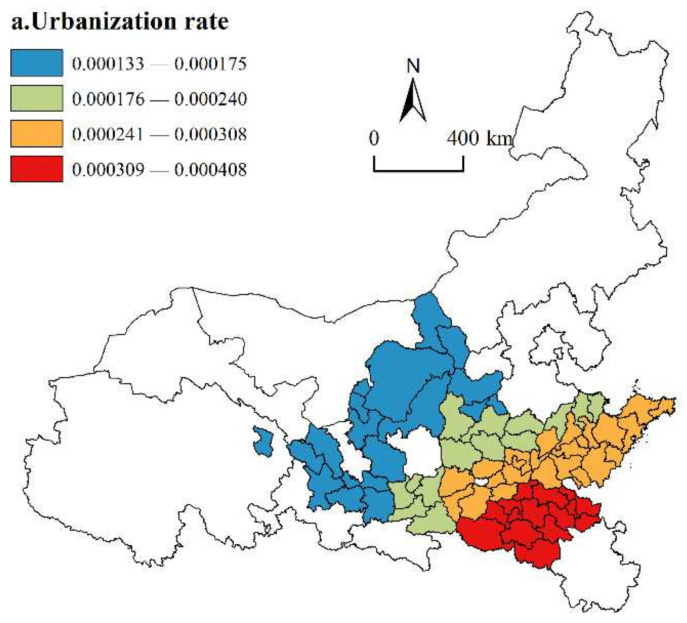
Spatial distribution of urbanization rate.

As shown in Figure 7, the regression coefficients of the population urbanization rate are all positive, indicating that improving the population urbanization rate is an important way to promote the HQD of urban agglomerations along the YRB. Increasing the urbanization rate of the population provides more rural people with opportunities to participate in socialist modernization construction and accelerates the HQD of urban agglomerations. In terms of the spatial distribution, the regression coefficients show a multi-banded spatial pattern increasing from northwest to southeast. The high-value regression coefficient areas are located in the southern areas of the Central Plains urban agglomeration (Huaibei, Bengbu, Pingdingshan, etc.), which have a dense population and high urbanization level, can boost consumer demand, form consumption capacity and an active market economy, and promote the positive development of urban agglomerations at a high level. The zones with low regression coefficient values are located in three upstream urban agglomerations and some cities in two midstream urban agglomerations. In the upstream urban agglomerations, only the core cities of Ningxia, Lanzhou and Xining have the same urbanization rate as the whole of China, while the other cities are obviously lower. The foundation of urbanization in these areas has difficulty supporting the further development of urban agglomerations. In the middle reaches, although the urbanization rate has reached or even exceeded the urbanization level of China, under the two-way influence of the development advantages of the lower reaches and the preferential policies of the upper reaches, the urbanization development momentum of the population in the middle reaches is insufficient. In contrast, HQD must provide a continuous stream of high-quality urbanization populations. This factor plays a limited role in helping the seven urban agglomerations achieve higher development levels.

(2) Industrial structure upgrading index

**Figure 8:**
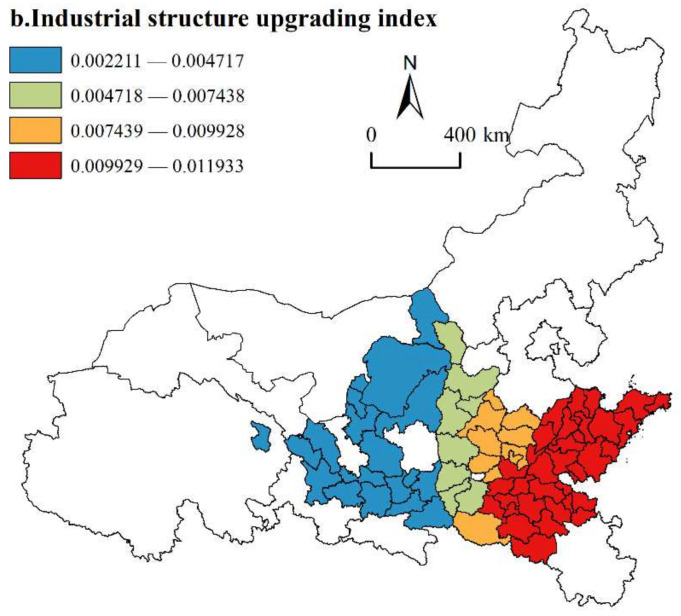
Spatial distribution of industrial structure upgrading index.

As shown in Figure 8, the regression coefficients of the industrial structure upgrading index are all positive, which indicates that upgrading the indexes of advanced industrial structure has an important driving effect on the HQD of urban agglomerations along the YRB. With the development of advanced industrial structures, the primary industry gradually evolves into secondary and tertiary industries, and labor-intensive industries gradually evolve into capital-intensive and technological knowledge-intensive industries. As industrial productivity and income have greatly improved, the urban economy has changed. In terms of spatial distribution, the results of industrial structure upgrading show strong spatial heterogeneity, and the regression coefficients increase from west to east. The areas with high regression coefficients are covered by the Shandong Peninsula and the Central Plains Urban Agglomerations in the lower reaches of the YRB. The Shandong Peninsula Urban Agglomeration is located on the southeast coast, with superior geographical location and convenient transportation. It was given priority to develop industry since the reform and opening-up. The Central Plains Urban Agglomeration has a good foundation for the manufacturing industry. With the accumulation of talent and the economy, urban agglomerations further define the development direction of industrial structures. By strengthening advanced manufacturing clusters, cultivating strategic emerging industrial clusters, and speeding up the modern service industry, the government can continuously promote the transformation and upgrade of the industrial structure and accelerate the HQD of urban agglomerations. However, upstream urban agglomerations are still dominated by traditional industries, and the industrial structure is not reasonable, resulting in large resource consumption and serious ecological damage, which has little effect on promoting the HQD of urban agglomerations.

(3) Proportion of R&D expenditure

**Figure 9:**
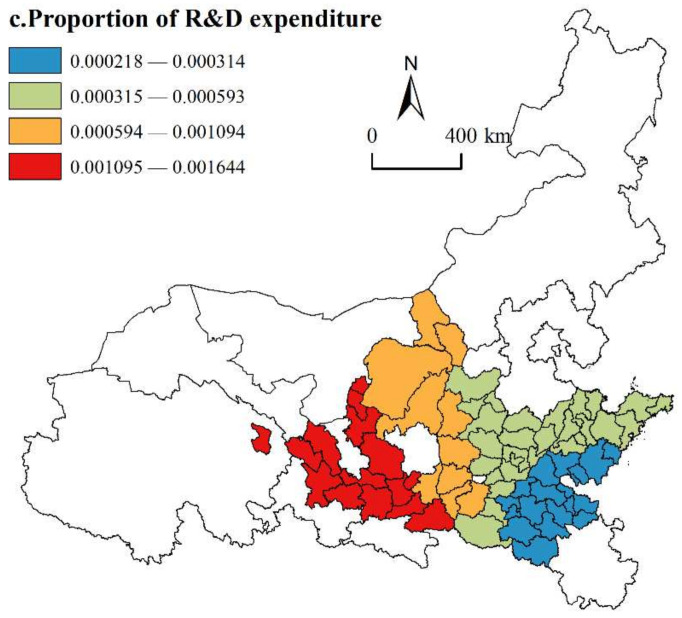
Spatial distribution of proportion of R&D expenditure.

As shown in Figure 9, the regression coefficients of the proportion of R&D expenditure are all positive, which indicates that increasing the investment of scientific research funds can help urban agglomerations along the YRB achieve HQD. Innovation investment can attract innovative talent, increase innovation platforms, stimulate innovation vitality, and help urban agglomerations achieve innovation development. In terms of spatial distribution, the proportion of R&D investment in the seven urban agglomerations has a massive effect, and the driving effect gradually appears from southeast to southwest. The areas with high regression coefficient values are located in some cities of the Lanxi Urban Agglomeration, Ningxia Urban Agglomeration and Guanzhong Plain Urban Agglomeration. Compared with other urban agglomerations in the basin, the upstream urban agglomerations are limited by the fragile ecological environment and a weak economy, and the R&D expenditure level is relatively low. However, in recent years, with the implementation of the “Western Development” and the “Belt and Road” strategies, the HQD of upstream urban agglomerations has been highly valued by the state. Therefore, the upstream urban agglomerations grasp the historical opportunity, and the Lanxi Urban Agglomeration speeds up the construction of the “Minhe-Honggu” innovation and development Pioneer Area. The Ningxia Urban Agglomeration starts from the “Ningdong base”, deeply implements an innovation-driven development strategy, and actively creates new heights for innovation to push the economy and society forward, injecting inexhaustible power into the development of the urban agglomeration. The areas with low regression coefficient values are located in the Shandong Peninsula Urban Agglomeration (Rizhao, Zaozhuang, Heze, etc.) and some cities of the Central Plains Urban Agglomeration (Bozhou, Xuchang, Xinyang, etc.), which belong to the third- or fourth-tier cities. Faced with the development pressure of the Matthew effect being greater than the diffusion effect of core cities in the cluster, these areas have deficiencies in innovation subjects, innovation platforms, innovation environments, etc. It has not yet formed the necessary innovation system support for the development and has little impact on the HQD of urban agglomerations.

(4) Internet penetration rate

**Figure 10:**
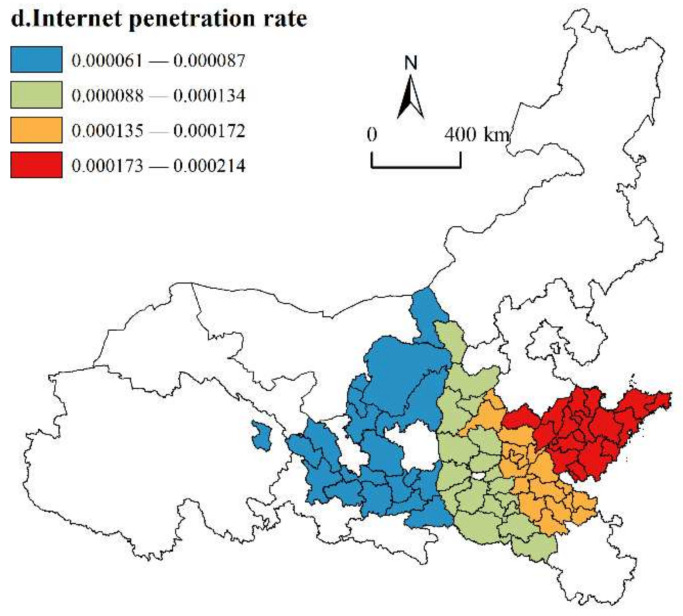
Spatial distribution of internet penetration rate.

As shown in Figure 10, the regression coefficients of the internet penetration rate are all positive, which indicates that improving the internet penetration rate is helpful for achieving the HQD of urban agglomerations along the YRB. With the improvement of the internet penetration rate, the interconnection among cities and urban agglomerations can be better realized, which has a strong significance to narrow the gap between urban–rural areas, solve the problem of urban–rural dual structure, and help urban agglomerations move towards HQD. In terms of spatial distribution, the regression coefficients show an increasing trend from west to east. The high value of the regression coefficient is located in the Shandong Peninsula Urban Agglomeration. The internet penetration rate of cities in this cluster is higher than the total mean value, laying a foundation for intercity connectivity. For example, Qingdao, as the economic center of Shandong Province, has realized the mutual development between urban–rural areas from the four aspects of “internet + industry”, “internet + service industry”, “internet + agriculture” and “internet + government affairs”, which have a remarkable impact on the overall HQD of urban agglomeration. The low-value areas of the regression coefficient are located in three urban agglomerations in the upper reaches and some cities in the Guanzhong Plain Urban Agglomerations (Pingliang, Qingyang, Tongchuan, etc.). These areas have not yet formed a complete information network system, and it is difficult to obtain more satisfactory economic benefits in exchange for the excessive payment consideration required to promote information resources and sharing services. As a result, the development of the internet economy is slow, which delays the development speed and efficiency and thus has little effect on the HQD of urban agglomerations.

(5) Proportion of the urban construction area

**Figure 11:**
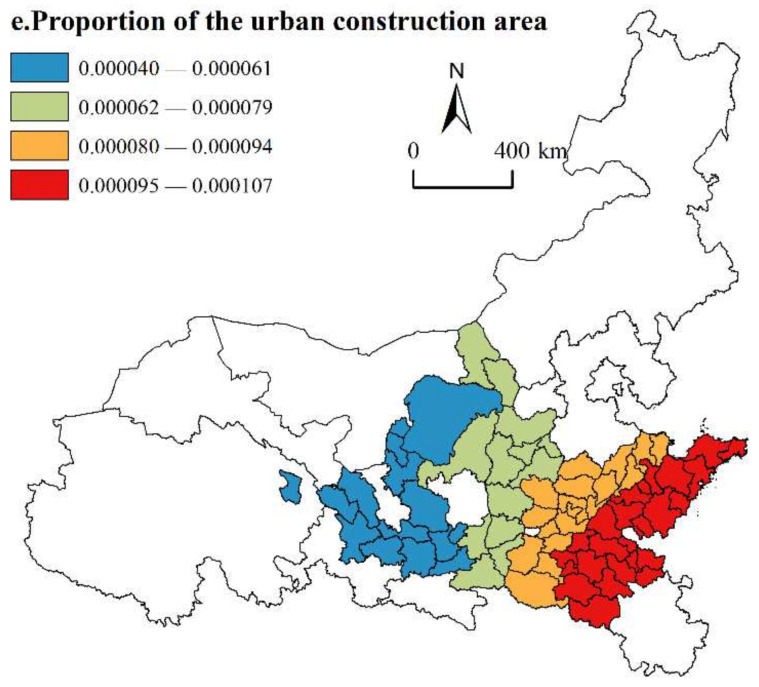
Spatial distribution of proportion of the urban construction area.

As shown in Figure 11, the regression coefficients of the urban construction area proportion are also positive, indicating that active and reasonable urban construction has a positive impact on promoting the HQD of urban agglomerations along the YRB. The moderate total amount and optimized structure of urban construction land are conducive to creating efficient urban production space and liveable urban living space and help urban agglomerations produce and create. In terms of the spatial distribution of urban construction, the effect of expanding urban construction area on the HQD of seven urban agglomerations has a hierarchical zonal distribution, and the driving effect gradually appears from northwest to southeast. The areas with high regression coefficient values are located in some cities in the Shandong Peninsula Urban Agglomeration (Jining, Qingdao, Heze, etc.) and Central Plains Urban Agglomeration (Xinyang, Zhoukou, Zhumadian, etc.). These two urban agglomerations are regional urban agglomerations. Compared with other local urban agglomerations along the YRB, they have larger populations, closer economic ties and broader market development. The reasonable expansion of urban construction has played a more positive impact in boosting the development of the urban agglomeration. The areas with low regression coefficient values are located in three urban agglomerations in the upper reaches and in some cities in the Guanzhong Plain Urban Agglomerations (Tongchuan, Baoji, Xianyang, etc.). The upstream urban agglomerations are vast and sparsely populated. Even if urban construction is expanded, the lack of investment in population, industry and innovation elements will also play a limited role in driving the development of urban agglomerations. The Guanzhong Plain Urban Agglomeration should take the structure of land use into consideration to prevent the uncoordinated and disorderly expansion of urban construction land, causing the inefficiency of urban construction land to occur, and cannot promote the development of urban agglomeration.

(6) Proportion of days reaching air standard

**Figure 12:**
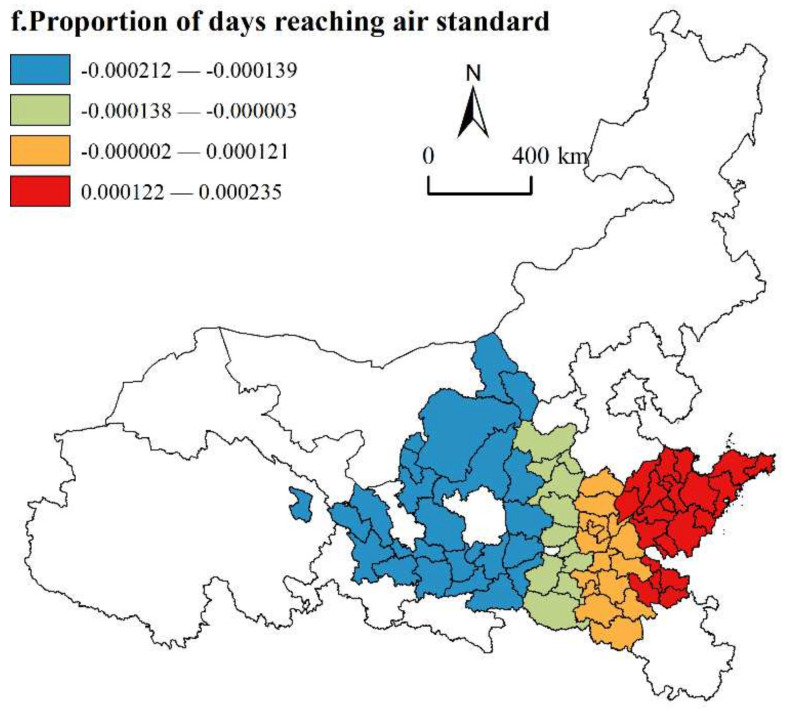
Spatial distribution of proportion of days reaching air standard.

As shown in Figure 12, the proportion of days reaching the air standard has a two-way influence on the HQD of urban agglomerations along the YRB, and the negative influence is distributed in the middle and upper reaches of the YRB. This region is the most fragile ecological environment in the whole YRB, and it is difficult to repair the ecological environment damage caused by the irrational industrial structure in this region, which further hinders the pace of HQD. As the central city of the Lanxi Urban Agglomeration, Gansu, the imbalance of industrial structure and the decline of input–output efficiency make resource consumption rise sharply, and the reduction in the pollution control level weakens the driving effect of institutional innovation and technological innovation on HQD. The adjacent Ningxia Urban Agglomeration consumes many eco-environmental resources, and the promotion power is seriously insufficient, so it is difficult to coordinate the ecological environment with HQD. The regression coefficients of most cities in the Shandong Peninsula Urban Agglomeration and Central Plains Urban Agglomeration in the lower reaches of the YRB are positive. Improving the ambient air quality can reduce pollutant emissions, optimize the atmospheric environment, and have a positive health impact on the human production life ecosystem. Among them, the Shandong Peninsula Urban Agglomeration should give full play to its geographical advantages and highlight the protection of mountains, rivers, coasts, wetlands and other important ecological environments. By carrying out green, circular, and low-carbon development to promote the development of ecological resources, ecological environment protection and industrial structure transformation and upgrading, the overall level of the regional ecological environment has been greatly improved, which has a positive role in the HQD of urban agglomerations.

## 4. Conclusions

Based on the panel data of 70 cities in the urban agglomerations along the YRB from 2009 to 2018, this paper constructs a high-quality evaluation system to analyze the spatiotemporal characteristics of the HQD levels of seven urban agglomerations and uses GWR to explore the spatial heterogeneity of the influencing factors of the HQD of the urban agglomerations along the YRB. The following conclusions are obtained.

First, over the past 10 years, the HQD level of the Shandong Peninsula Urban Agglomeration was far ahead; at the same time, the gap among the seven urban agglomerations was gradually narrowing. From the perspective of temporal change, the HQD levels of the Guanzhong Plain Urban Agglomeration and Central Plains Urban Agglomeration have increased significantly, while those of the Shandong Peninsula Urban Agglomeration and Ningxia Urban Agglomeration have decreased. From the perspective of spatial distribution, the HQD level of urban agglomerations along the YRB presents a distribution state of “high in the east, low in the west, depression in the middle”, and the HQD of cities in the cluster shows an obvious spatial pattern of “center-peripheral”.

Second, the spatial correlation of the HQD of the seven urban agglomerations is strong, and the agglomeration effect is obvious. The results of the local $G_{i}^{*}$ index show that the pattern of cold and hot spots of urban HQD along the YRB has not changed substantially, showing the characteristics of a few hot spots and many cold spots, roughly showing the spatial distribution of “hot in the east and cold in the west”. However, with the contraction of cold spots and hot spots and the expansion of sub-cold spots and sub-hot spots, the gap in HQD of the seven urban agglomerations is gradually narrowing.

Third, the driving factors have significant regional differences in their effect on the HQD of the seven urban agglomerations. In general, the influence degree of six driving forces ranked as follows: industrial structure upgrading index > proportion of R&D expenditure > urbanization rate > internet penetration rate > proportion of urban construction area > proportion of days reaching the air standard, which indicates that advanced industrial structure and technology are the two core driving forces for the HQD of urban agglomerations along the YRB. Only the proportion of days reaching the air standard shows positive and negative effects, the fragile ecological environment hinders the pace of high-quality development, and the improved ecological environment helps the HQD of urban agglomerations. The other variables show significant positive effects. Improving the urbanization rate of the population, upgrading the level of industrial structure, increasing investment in scientific research, improving the internet penetration rate, and actively and reasonably urban construction has a positive impact on promoting the HQD of urban agglomerations in the YRB. Among them, the driving factors play an important role in the middle and lower reaches of the YRB.

## 5. Recommendations

First, the process of urbanization should be promoted. The results show that population urbanization is an important way to promote the HQD of urban agglomerations in the YRB. For upstream urban agglomerations with low urbanization levels and less promotion, it is necessary to strengthen the construction of infrastructure to prevent the excessive outflow of population. At the same time, we should optimize social security policies to enhance the attractiveness of urban agglomerations and guide the steady growth of the population further. We can also build a good ecological environment, improve the liveability of the city, and promote the appropriate concentration of population in the group. For the lower reaches of the Shandong Peninsula Urban Agglomeration, which has a high level of urbanization but has not been brought into full play, we should, according to the characteristics of resources and environment carrying capacity and productivity distribution, avoid the adverse effects of excessive population concentration, reasonably guide the orderly transfer of rural population to cities, actively guide the evacuation of industries and infrastructure in urban densely populated areas to the surrounding areas of big cities, and promote the coordinated development of the urban population in the upper and lower reaches.

Second, the industrial structure should be optimized. The results show that the advanced level of the industrial structure plays an important driving role in the HQD evolution of urban agglomerations in the YRB. Therefore, on the one hand, we should promote the rationalization of the industrial structure. For upstream urban agglomerations, we should be based on resource endowments and industrial foundations, with green, efficient, clean and low carbon and cultivated and expanded green emerging industries. For downstream urban agglomerations, we should accelerate the transformation and upgrading of traditional industries, highlight the cultivation of emerging industries and new forms of business, undertake industrial transfer in an orderly manner, and build a modern industrial system with complementary advantages, close cooperation and linkage development. On the other hand, we should promote the upgrading of the industrial structure. Hence, each urban agglomeration should make the overall quality and efficiency of the industrial structure evolve to a higher level through technological progress. For downstream cities with a good industrial foundation, innovation should be taken as the driving force to promote the transformation and upgrading of traditional industries. By promoting industry-city integration and establishing an industrial cooperation mechanism, we strive to build a modern industrial system with horizontally dislocated development and a vertical division of labor and cooperation.

Third, expand the investment in scientific research so as to improve the level of urban innovation. It can be seen from the results that improving the innovation ability of urban agglomerations contributes to the HQD of urban agglomerations in the YRB. However, it is necessary to narrow the regional gap of urban agglomeration innovation ability and eliminate the polarization phenomenon of urban agglomeration innovation ability [47]. (1) We should attach importance to the regional coordinated development strategy that has urban agglomeration as the core and create a new growth pole of economic development under the new normal. (2) A coordination mechanism for innovation ability must be established quickly in the seven urban agglomerations. Policy, capital, technology, education and other aspects promote the innovation ability of urban agglomerations, optimize the innovation environment, promote the flow of talent, and realize the coordinated development of urban agglomerations.

Fourth, increase internet penetration, so as to enhance the level of urban innovation. The improvement of the informatization level helps to strengthen the interconnection between urban agglomeration [48]. The results show that the infrastructure construction of interconnectivity is an effective way to improve the level of regional HQD and should be prioritized in planning. In terms of planning, the coverage of intercity railways should be improved, the traffic capacity for the construction of traffic infrastructure should be enhanced, and full coverage and wireless local area networks (WLANs) should be realized in key areas and key lines. In terms of capital, it is necessary to clarify the guiding role of provincial financial funds and the leading role of urban funds and establish special funds for the development of urban public transportation. At the same time, an investment fund for the integrated development of urban agglomerations should be set up, and private capital should be encouraged to participate in the establishment and operation of the fund. In terms of operation, the first is to improve the transport service capacity and level. Implementation of key projects, such as “Internet+” convenient transportation. Then, we should improve inclusive information services, steadily reduce telecommunications charges, and strengthen the security protection of information infrastructure and information resources.

Fifth, the rational layout of urban agglomeration space should be promoted. The HQD of urban agglomerations depends on the joint action of accurate development planning, reasonable spatial layout and efficient regional coordination mechanism. (1) Precise development planning requires the local government to comprehensively grasp the local economic development situation. (2) The reasonable spatial layout of urban agglomerations led by functional orientation should be promoted by improving the extension elasticity of geographical space. For upstream urban agglomerations with good resource endowments but fragile ecological environments, it is essential to adjust the spatial structure, improve the utilization efficiency of urban space, and actively build a spatial pattern suitable for the carrying capacity of resources and the environment according to the development principle of land space suitability and resources and the environment carrying capacity. (3) An important obstacle to the harmonious development of the seven studied urban agglomerations is the poor regional communication and coordination caused by administrative segmentation. Therefore, we can promote the rational distribution of regional space by innovating regional coordination mechanisms.

Sixth, we will increase the number of days when the air reaches the standard, and promote joint construction, joint protection and joint governance of the ecological environment should be promoted. Generally, the development level of the ecological environment lags behind the new urbanization in the YRB. (1) Protection should be given priority. The upstream urban agglomerations should strengthen the control of forests, grasslands, rivers, lakes, beaches, coastlines, oceans, deserts, mountains and other important ecological spaces. The midstream urban agglomerations should scientifically implement the protection and restoration projects of important ecological resources such as water, lakes, mountains, forests, fields and grasses; The downstream urban agglomerations should persist in adhering to the concept of green, low-carbon and sustainable development, and establish and improve the linkage mechanism of cross-regional ecological civilization construction. (2) Governance should be strengthened. An emergency early warning mechanism for regional air pollution can be established to strengthen the network construction of soil environmental quality monitoring and strengthen the joint control of air pollution, water pollution and soil pollution in river basins. (3) Green development should be promoted. To this end, the seven urban agglomerations along the YRB should improve the ecological civilization system, improve the efficiency of resource utilization, strengthen energy conservation, emission reduction and carbon reduction, develop the urban circular economy, and advance the green and low-carbon development of urban agglomerations.

## Figures and Tables

**Table 1:** Evaluation index system for HQD of urban agglomerations.

Target Layer	Criterion Layer	Index Layer	Unit	Attribute	Entropy Weight in 2009	Entropy Weight in 2018
Innovative development	Innovation input	X1: Proportion of science expenditure in the general public budget	%	+	0.0369	0.0357
		X2: Proportion of education expenditure in the general public budget	%	+	0.0214	0.0305
	Innovation output	X3: Number of patents granted per 10,000 people	1	+	0.0978	0.0381
Coordinated development	Regional coordination	X4: Proportion of regional GDP	%	+	0.0537	0.0500
		X5: Per capita disposable income of urban residents	10,000 CNY	+	0.0435	0.1031
	Urban and rural coordination	X6: Urban–rural income ratio	%	−	0.0276	0.0336
		X7: Urban–rural consumption ratio	%	−	0.0271	0.0217
Green development	Urban greening	X8: Green coverage rate in built-up areas	%	+	0.0343	0.0223
		X9: Acreage of park and greenbelt	$m^{2}$	+	0.0768	0.0793
	Pollution discharge	X10: Wastewater discharge per 10,000 CNY of industrial output value	ton	−	0.0206	0.0268
		X11: $\;SO_{2}\;$emissions per 10,000 CNY of industrial output value	ton	−	0.0266	0.0303
		X12: Smoke and dust emissions per 10,000 CNY of total industrial output value	ton	−	0.0262	0.0252
	Green governance	X13: Treatment rate of urban domestic sewage	%	+	0.0328	0.0198
		X14: Harmless disposal rate of garbage	%	+	0.0413	0.0185
		X15: Comprehensive utilisation rate of industrial solid waste	%	+	0.0419	0.0374
Opening development	Trade opening	X16: Foreign trade dependence	%	+	0.0665	0.0903
	Investment opening	X17: Foreign capital dependence	%	+	0.0368	0.0599
	Tourism opening	X18: Proportion of total tourism income	%	+	0.0513	0.0447
		X19: Proportion of arrivals	%	+	0.0425	0.0437
Sharing development	Urban roads	X20: Actual urban road area at the end of the year	${\mathrm{hm}}^{2}$	+	0.0686	0.0552
	Public transportation	X21: Proportion of highway passenger traffic in total population	%	+	0.0503	0.0475
	Cultural sharing	X22: Volume of books in public libraries	Volume	+	0.0493	0.0540
	Medical security	X23: Number of doctors	person	+	0.0262	0.0325

+: The higher the proportion of science expenditure in the general public budget, the more cities pay attention to innovation, and the higher the innovation index; −: The higher the urban-rural income ratio, the greater the income gap between urban and rural areas, which is not conducive to the coordinated development of urban and rural areas.

**Table 2:** Moran’s I of HQD of seven urban agglomerations.

Year	Moran’s I	E(I)	V(I)	Z(I)	P(I)
2009	0.1004	−0.0145	0.0002	8.0809	0.0000
2010	0.0957	−0.0145	0.0002	7.7210	0.0000
2011	0.0906	−0.0145	0.0002	7.3606	0.0000
2012	0.0799	−0.0145	0.0002	6.6130	0.0000
2013	0.0722	−0.0145	0.0002	6.0690	0.0000
2014	0.0481	−0.0145	0.0002	4.4079	0.0000
2015	0.0464	−0.0145	0.0002	4.2911	0.0000
2016	0.0437	−0.0145	0.0002	4.1296	0.0000
2017	0.0389	−0.0145	0.0002	3.7712	0.0002
2018	0.0371	−0.0145	0.0002	3.6394	0.0003

**Table 3:** Driving factors of HQD of seven urban agglomerations.

Driving Factors	Index	Formula
Population size	Urbanisation rate	Urban population/total population
Industrial structure	Indexes of advanced industrial structure	Tertiary industry/secondary industry
Science and technology	Ratio of R&D expenditure	R&D expenditure/GDP
Informatisation level	Internet penetration rate	Number of internet users/total number of households at the end of the year
Urban construction	Proportion of urban construction area	Urban construction land area/urban area
Environmental quality	Proportion of days reaching air standard	Air standard days/360 days

**Table 4:** Results of multicollinearity test.

Variable	VIF	T	CI
Urbanisation rate	1.783	0.561	3.386
Industrial structure upgrading index	1.116	0.896	3.575
Proportion of R&D expenditure	1.485	0.673	7.055
Internet penetration rate	1.727	0.579	13.247
Proportion of urban construction area	1.332	0.751	17.684
Proportion of days reaching air standard	1.331	0.751	21.999

## Data Availability

The data presented in this study are openly available in the China Statistical Yearbook, China City Statistical Yearbook, China Statistical Yearbook for Regional Economy, EPS database, statistical yearbooks of relevant provinces and cities, statistical bulletins of national economic and social development.

## References

[1] Jiang W., Gao W., Gao X., Ma M., Zhou M., Du K., Ma X. (2021). Spatio-temporal heterogeneity of air pollution and its key influencing factors in the Yellow River Economic Belt of China from 2014 to 2019. J. Environ. Manag..

[2] Fang C.L. (2020). Spatial organization pattern and high quality development of urban agglomeration in the Yellow River Basin. Econ. Geogr..

[3] Ma H.T., Xu S.F. (2020). High quality development evaluation and spatial pattern differentiation of urban agglomerations in the Yellow River Basin. Econ. Geogr..

[4] Wohlfart C., Kuenzer C., Chen C., Liu G. (2016). Social-ecological challenges in the Yellow River basin (China): A review. Environ. Earth Sci..

[5] Howard E. (1898). Tomorrow: A Peaceful Path to Real Reform.

[6] Geddes P. (1915). Cities in Evolution.

[7] Gottmann J. (1957). Megalopolis or the urbanization of the northeastern seaboard. Econ. Geogr..

[8] Schneider A., Mertes C.M. (2014). Expansion and growth in Chinese cities, 1978–2010. Environ. Res. Lett..

[9] Heng Q. (2018). Navigating China’s Economic Development in the New Era from High-Speed to High-Quality Growth. China Q. Int. Strateg. Stud..

[10] Gu W., Wang J., Hua X., Liu Z. (2021). Entrepreneurship and high-quality economic development: Based on the triple bottom line of sustainable development. Int. Entrep. Manag. J..

[11] Zhou B., Zeng X., Jiang L., Xue B. (2020). High-quality Economic Growth under the Influence of Technological Innovation Preference in China: A Numerical Simulation from the Government Financial Perspective. Struct. Chance Econ. Dyn..

[12] Chen L., Ye W., Huo C., James K. (2020). Environmental Regulations, the Industrial Structure, and High-Quality Regional Economic Development: Evidence from China. Land.

[13] Huang X.H., Cai B.Q., Li Y.L. (2020). Evaluation Index System and Measurement of High-quality Development in China. Rev. Cercet. Si Interv. Soc..

[14] Li B., Tian C., Shi Z., Han Z. (2020). Evolution and Differentiation of High-Quality Development of Marine Economy: A Case Study from China. Complexity.

[15] Chen B., Chen G.Q. (2009). Emergy-based energy and material metabolism of the Yellow River basin. Commun. Nonlinear Sci. Numer. Simul..

[16] Feng M., Guo H.X. (2019). Research on the Evaluation of High-Quality Economic Development Based on Factor Analysis. J. Sci. Ind. Res..

[17] Chen Y., Zhu M., Lu J., Zhou Q., Ma W. (2020). Evaluation of ecological city and analysis of obstacle factors under the background of high-quality development: Taking cities in the Yellow River Basin as examples. Ecol. Indic..

[18] Fang C.L., Yu D.L. (2017). Urban agglomeration: An evolving concept of an emerging phenomenon. Landsc. Urban Plan..

[19] Zheng S.Q., Du R. (2020). How does urban agglomeration integration promote entrepreneurship in China? Evidence from regional human capital spillovers and market integration. Cities.

[20] Niu F.Q., Yang X.Y., Wang F. (2020). Urban Agglomeration Formation and Its Spatiotemporal Expansion Process in China: From the Perspective of Industrial Evolution. Chin. Geogr. Sci..

[21] Sun Y., Cui Y. (2018). Evaluating the coordinated development of economic, social and environmental benefits of urban public transportation infrastructure: Case study of four Chinese autonomous municipalities. Transp. Policy.

[22] Jiang X., Fu W., Li G.L. (2020). Can the improvement of living environment stimulate urban Innovation?—Analysis of high-quality innovative talents and foreign direct investment spillover effect mechanism. J. Clean. Prod..

[23] Jahanger A. (2021). Influence of FDI characteristics on high-quality development of China’s economy. Environ. Sci. Pollut. Res..

[24] Lu H.M., Zhang M.L., Sun W.W. (2018). Expansion Analysis of Yangtze River Delta Urban Agglomeration Using DMSP/OLS Nighttime Light Imagery for 1993 to 2012. ISPRS Int. J. Geo-Inf..

[25] Zhang W.J., Wang M.Y. (2018). Spatial-temporal characteristics and determinants of land urbanization quality in China: Evidence from 285 prefecture-level cities. Sustain. Cities Soc..

[26] Lu B., Brunsdon C., Charlton M., Harris P. (2017). Geographically weighted regression with parameter-specific distance metrics. Int. J. Geogr. Inf. Sci..

[27] Zhang H.P., Li M.H., Zeman Z. (2019). Study on Chinese technical economy and global social responsibility. J. Phys. Conf. Ser..

[28] Hu H.Q., Ma Y., Wu S.J. (2020). Fuzzy comprehensive evaluation on high-quality development of China’s rural economy based on entropy weight. J. Intell. Fuzzy Syst..

[29] Fan Y.P., Fang C.L., Zhang Q. (2019). Coupling coordinated development between social economy and ecological environment in Chinese provincial capital cities-assessment and policy implications. J. Clean. Prod..

[30] Chen J., Zhou Q. (2017). City size and urban labor productivity in China: New evidence from spatial city-level panel data analysis. Econ. Syst..

[31] He Y., Choi B.R. (2020). China’s Outward Foreign Direct Investment Patterns: Evidence from Asian Financial Markets. J. Asia Financ. Econ. Bus..

[32] Jiang J., Luo L., Xu P., Wang P. (2018). How does social development influence life expectancy? A geographically weighted regression analysis in China. Public Health.

[33] Wei C.Z., Taubenbock H., Blaschke T. (2017). Measuring urban agglomeration using a city-scale dasymetric population map: A study in the Pearl River Delta, China. Habitat Int..

[34] Shen Y.L., Yao L. (2017). PM2.5, Population Exposure and Economic Effects in Urban Agglomerations of China Using Ground-Based Monitoring Data. Int. J. Environ. Res. Public Health.

[35] Han F., Xie R., Fang J.Y. (2018). Urban agglomeration economies and industrial energy efficiency. Energy.

[36] Li Z., Sun L., Geng Y., Dong H., Ren J., Liu Z., Tian X., Yabar H., Higano Y. (2017). Examining industrial structure changes and corresponding carbon emission reduction effect by combining input-output analysis and social network analysis: A comparison study of China and Japan. J. Clean. Prod..

[37] Liu K., Jiang H., Zhou Q. (2021). Spatial Analysis of Industrial Green Development and Sustainable Cities in the Yellow River Basin. Discret. Dyn. Nat. Soc..

[38] Jokanović B., Lalic B., Milovančević M., Simeunović N., Marković D. (2017). Economic development evaluation based on science and patents. Phys. A-Stat. Mech. Appl..

[39] Chen J., Wei H., Li N., Chen S., Qu W., Zhang Y. (2020). Exploring the Spatial-Temporal Dynamics of the Yangtze River Delta Urban Agglomeration Based on Night-Time Light Remote Sensing Technology. IEEE J. Sel. Top. Appl. Earth Obs. Remote Sens..

[40] Yu X., Wu Z., Zheng H., Li M., Tan T. (2020). How urban agglomeration improve the emission efficiency? A spatial econometric analysis of the Yangtze River Delta urban agglomeration in China. J. Environ. Manag..

[41] Xiong Y., Chen Y., Peng F., Li J., Yan X. (2019). Analog simulation of urban construction land supply and demand in Chang-Zhu-Tan Urban Agglomeration based on land intensive use. J. Geogr. Sci..

[42] Li Z., Jiang W., Wang W., Lei X., Deng Y. (2019). Exploring spatial-temporal change and gravity center movement of construction land in the Chang-Zhu-Tan urban agglomeration. J. Geogr. Sci..

[43] Sun J., Li Y.P., Gao P.P., Xia B.C. (2018). A Mamdani fuzzy inference approach for assessing ecological security in the Pearl River Delta urban agglomeration, China. Ecol. Indic..

[44] He C., Chen T., Mao X., Zhou Y. (2016). Economic transition, urbanization and population redistribution in China. Habitat Int..

[45] Ertugrul H.M., Cetin M., Seker F., Dogan E. (2016). The impact of trade openness on global carbon dioxide emissions: Evidence from the top ten emitters among developing countries. Ecol. Indic..

[46] Sun J., Wang J., Wang T., Zhang T. (2019). Urbanization, economic growth, and environmental pollution Partial differential analysis based on the spatial Durbin model. Manag. Environ. Qual..

[47] Su Z., Wen G., Rui H. A Comparative Study of Polarization Effects of Regional Innovation in China—Calculation Based on TW Index. Proceedings of the 4th International Conference on Social Science and Higher Education (ICSSHE).

[48] Wang D., Zhou T., Wang M.M. (2021). Information and communication technology (ICT), digital divide and urbanization: Evidence from Chinese cities. Technol. Soc..

